# Emergent Subpopulation Behavior Uncovered with a Community Dynamic Metabolic Model of *Escherichia coli* Diauxic Growth

**DOI:** 10.1128/mSystems.00230-18

**Published:** 2019-01-15

**Authors:** Antonella Succurro, Daniel Segrè, Oliver Ebenhöh

**Affiliations:** a Botanical Institute, University of Cologne, Cologne, Germany; b Cluster of Excellence on Plant Sciences (CEPLAS), Düsseldorf, Germany; c Bioinformatics Program and Biological Design Center, Boston University, Boston, Massachusetts, USA; d Department of Biology, Department of Biomedical Engineering, Department of Physics, Boston University, Boston, Massachusetts, USA; e Institute for Quantitative and Theoretical Biology, Heinrich Heine University, Düsseldorf, Germany; U.S. Naval Research Laboratory

**Keywords:** diauxic growth, metabolic network modeling, microbial communities, population heterogeneity

## Abstract

Microbes have adapted to greatly variable environments in order to survive both short-term perturbations and permanent changes. A classical and yet still actively studied example of adaptation to dynamic environments is the diauxic shift of Escherichia coli, in which cells grow on glucose until its exhaustion and then transition to using previously secreted acetate. Here we tested different hypotheses concerning the nature of this transition by using dynamic metabolic modeling. To reach this goal, we developed an open source modeling framework integrating dynamic models (ordinary differential equation systems) with structural models (metabolic networks) which can take into account the behavior of multiple subpopulations and smooth flux transitions between time points. We used this framework to model the diauxic shift, first with a single E. coli model whose metabolic state represents the overall population average and then with a community of two subpopulations, each growing exclusively on one carbon source (glucose or acetate). After introduction of an environment-dependent transition function that determined the balance between subpopulations, our model generated predictions that are in strong agreement with published data. Our results thus support recent experimental evidence that diauxie, rather than a coordinated metabolic shift, would be the emergent pattern of individual cells differentiating for optimal growth on different substrates. This work offers a new perspective on the use of dynamic metabolic modeling to investigate population heterogeneity dynamics. The proposed approach can easily be applied to other biological systems composed of metabolically distinct, interconverting subpopulations and could be extended to include single-cell-level stochasticity.

**IMPORTANCE** Escherichia coli diauxie is a fundamental example of metabolic adaptation, a phenomenon that is not yet completely understood. Further insight into this process can be achieved by integrating experimental and computational modeling methods. We present a dynamic metabolic modeling approach that captures diauxie as an emergent property of subpopulation dynamics in E. coli monocultures. Without fine-tuning the parameters of the E. coli core metabolic model, we achieved good agreement with published data. Our results suggest that single-organism metabolic models can only approximate the average metabolic state of a population, therefore offering a new perspective on the use of such modeling approaches. The open source modeling framework that we provide can be applied to model general subpopulation systems in more-complex environments and can be extended to include single-cell-level stochasticity.

## Abstract (precis)

Escherichia coli diauxie is a fundamental example of metabolic adaptation, a phenomenon that is not yet completely understood. Further insight into this process can be achieved by integrating experimental and computational modeling methods. We present a dynamic metabolic modeling approach that captures diauxie as an emergent property of subpopulation dynamics in E. coli monocultures. Without fine-tuning the parameters of the E. coli core metabolic model, we achieved good agreement with published data. Our results suggest that single-organism metabolic models can only approximate the average metabolic state of a population, therefore offering a new perspective on the use of such modeling approaches. The open source modeling framework that we provide can be applied to model general subpopulation systems in more-complex environments and can be extended to include single-cell-level stochasticity.

## INTRODUCTION

In natural environments, microorganisms are exposed to high fluctuations of nutrient and micronutrient availability and have therefore evolved adaptation strategies, both short term (to respond to temporary perturbations) and long term (to increase evolutionary fitness) (1). We still lack a sound theoretical understanding of the mechanisms driving such strategies, but the recent technological advances in high-throughput experimental techniques pave the way to novel approaches that integrate experimental and theoretical biology (2). Theoretical ecology describes ecosystems in mathematical terms as dynamic organism-environment interactions (3). As in statistical physics, individual behaviors in an ensemble result in observable emergent patterns that can be modeled with mathematical equations (4). This is the case for the earliest models of population dynamics developed by Verhulst (5), Lotka (6), and Volterra (7) and for the pioneering work of Jacques Monod in modeling microbial growth (8). With the rising academic and industrial interest in the “microbiome,” systems biology approaches are becoming a new standard (9) and more methods for the mathematical modeling of microbial communities are being developed (10, 11).

In constraint-based stoichiometric modeling, the metabolic network model of an organism is reconstructed from its annotated genome and described mathematically as a stoichiometric matrix (S). After imposition of the steady-state assumption and introduction of thermodynamic and biological boundaries for the metabolic fluxes ($\vec{v}$), flux balance analysis (FBA) (12, 13) defines an optimization problem in order to identify one particular flux distribution in the solution space. As long as the objective function (which imposes further biological assumptions on the system) is linear in the fluxes, the optimization problem can be solved by linear programming (LP). FBA returns a unique solution for the objective function, but the metabolic flux distribution is generally not unique, especially in genome-scale metabolic network models (GEMs). On the basis of the hypothesis that metabolism has evolved to make efficient use of resources and minimize waste, two specific methods were developed to extend FBA: parsimonious FBA (pFBA) (14) and minimization of metabolic adjustment (MOMA) (15). In pFBA, a second LP is defined such that the value of the objective function is set to the FBA solution and the new objective is the minimization of the overall fluxes. MOMA was developed to simulate the response to the perturbation introduced by gene deletion and is based on the principle that the organism would readjust its metabolism to a minimally different configuration with respect to the wild-type optimum. Another extension of FBA, dynamic FBA (dFBA) (16), allows partial recovery of the dynamic information lost under the conditions of the steady-state assumption. In the static optimization approach (SOA) that underlies dFBA, time is divided into discrete intervals and a new FBA problem is solved at time *t_i_* after updating of the external conditions according to the FBA solution at time *t_i_*_−1_. Approaches to modeling of microbial communities with GEMs have been recently reviewed by Succurro and Ebenhöh (17).

FBA and dFBA have been applied to the study of one of the most basic examples of metabolic transitions: diauxie (16, 18, 19). Discovered in the model organism Escherichia coli in 1941 by Monod (8, 20), diauxie remains a topic of active research (21–23). Under aerobic conditions with glucose as the sole carbon source (and generally the preferred one), E. coli secretes acetate during growth, which it then consumes once the glucose is exhausted. The molecular mechanisms driving this transition are still not completely understood, but over the last few years the fundamental roles of stochasticity and population heterogeneity have been demonstrated experimentally (24), often with the support of mathematical models. Indeed, in unpredictable natural environments fluctuating conditions of nutrient availability and variable fitness landscapes, homogeneous populations are more likely to face extinction, and bet hedging provides a selective advantage (25). Single-cell studies have suggested that the observed biphasic growth possibly represents the effect of stochastic gene expression (21), eventually coregulated by memory mechanisms (26). Kotte et al. (27) systematically investigated bistability in a clonal E. coli population. After ruling out responsive switching as a homogeneous adaptation, their results strongly suggested that the heterogeneous adaptation that results in two coexisting phenotypes was driven by responsive diversification (where a single phenotype diversifies in response to environmental changes) rather than stochastic switching (where the two phenotypes would coexist from the beginning). Although stochastic mathematical models have been proposed to support those findings, metabolic modeling approaches are only considered suitable to describe homogeneous systems, with single-organism GEMs representing the average population metabolic state.

Varma and Palsson (18) performed the first dFBA of E. coli, with a single GEM growing aerobically first on glucose and then on the secreted acetate. Here we present a study of E. coli diauxic growth on these two carbon sources, with the bacterial population modeled either as having an average, unique metabolic state (standard FBA and dFBA approach) or as being composed of two E. coli populations adapted to one of the two carbon sources. We used a modeling approach that integrates ordinary differential equation (ODE) models with dFBA, extending methods typically applied to study the dynamics of multispecies communities to the investigation of emergent patterns from individual behavior in monocultures. We implemented three approaches: (i) we modeled a homogeneous and yet smooth shift, with a single E. coli GEM, by adapting the MOMA algorithm; (ii) we introduced the hypothesis of subpopulations growing on specific carbon sources and model population transition as a purely stochastic mechanism; and (iii) we introduced an environment-driven response. Our results suggest that diauxie, rather than being modeled as a coordinated metabolic shift, can be modeled as the emergent pattern resulting from subpopulations optimizing growth on different substrates in response to environmental changes. This is much in agreement with experimental evidence from, e.g., Kotte et al. (27) and offers a new perspective on the use of dynamic metabolic modeling to investigate population dynamics. The proposed approach can easily be transferred to studies of generic subpopulations or communities and ultimately can be expanded to investigate single-cell dynamics.

## RESULTS

We ran simulations with an open source modeling framework developed to model ecosystem dynamics. The models are ODE systems solved with integrating routines that at each integration step solve an FBA problem. We first validated the E. coli GEM on the data from Varma and Palsson (18) (who reported the first dFBA of the glucose-acetate shift) and then used the calibrated model to reproduce the independent sets of experiments described by Enjalbert et al. (22) (who experimentally analyzed E. coli grown in aerobic batch systems with different concentrations of glucose and acetate). In the standard dFBA approach, a population is modeled with a unique GEM and fluxes instantaneously change to adapt to new environmental conditions. In reality, however, transcriptional changes and flux rerouting may cause delays which are not captured by existing algorithms. Furthermore, dFBA might predict metabolic states in which more carbon sources are simultaneously utilized, and it is not obvious that such an approach would correctly capture the complexity of a population diversifying into metabolically distinct subpopulations. Therefore, we modified the dFBA algorithm by taking advantage of optimization strategies previously developed for different biological issues and implemented novel concepts as well. In particular, we used either pFBA (14) or an adaptation of MOMA (15) to solve the FBA problem at each time step, replicating the standard dFBA approach or implementing a homogeneous and yet smooth shift, respectively. The MOMA algorithm was integrated into the dFBA routine by imposing the constraint that the solution of the FBA problem at time *t_i_* had to be minimally different from the solution at time *t_i−_*_1_. We tested three different hypotheses: (i) homogeneous, smooth population shift; (ii) stochastic population shift; and (iii) environment-driven subpopulation differentiation. We observed that dFBA performed with both pFBA and MOMA predicted abrupt transitions from acetate catabolism to acetate anabolism and that condition-specific parameterizations were necessary to reproduce the different data. We then modeled two E. coli subpopulations growing exclusively on glucose or acetate. For this, we extended the standard dFBA approach to include the process of population shifts. We tested whether purely stochastic switches (ii) or, rather, responsive diversification (iii) could capture the diauxic behavior by modeling the population transitions either with constant rates (ii) or with a heuristic function dependent on carbon source concentrations (iii). We observed that only model iii could reproduce data from different experiments with a unique set of parameters. We did not find significant improvements using MOMA rather than pFBA within the same metabolic state, so the simpler pFBA implementation was used in the subpopulation simulations where each model was fixed into one metabolic configuration. Further details of the modeling approach are provided in Materials and Methods.

### E. coli diauxie modeled with a uniform population.

**FIG 1:**
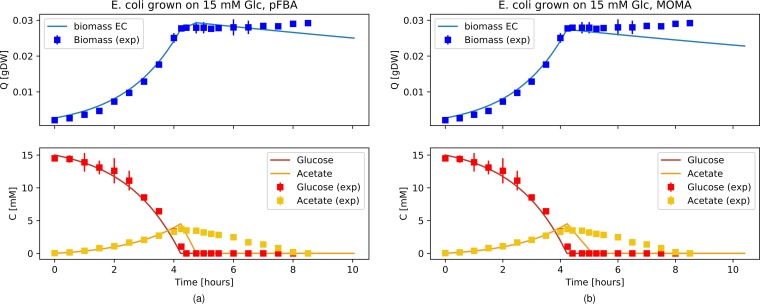
Diauxic growth of E. coli modeled as a uniform population under batch conditions. Simulation data (lines) are compared to data from Enjalbert et al. (22) (squares) as a function of time. Biomass data (blue, top subplots) and glucose and acetate data (red and yellow, bottom subplots) are shown. The flux distribution at each time step was obtained with pFBA (a) or MOMA (b). gDW, grams dry weight; C, concentration; Q, quantity.

**FIG 2:**
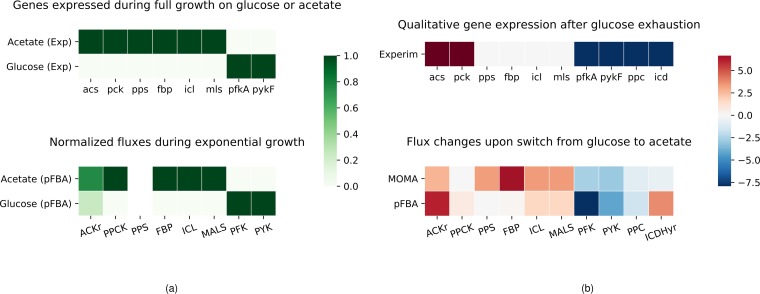
Comparisons of experimental information on gene expression levels with simulated flux distributions. The top plots qualitatively represent the gene expression data from Enjalbert et al. (22). Flux solutions in the simulations for the reactions associated with the reported key genes are compared between two independent simulations with E. coli exponentially growing either on acetate or on glucose (a) and within the same simulation (b) (growth on glucose simulated with MOMA or pFBA) (Fig. 1) before and after the point of glucose exhaustion. Genes: acs, acetyl coenzyme A synthetase; pck, phosphoenolpyruvate carboxykinase; pps, phosphoenolpyruvate synthetase; fbp, fructose-1,6-biphosphatase; icl, isocitrate lyase; mls, malate synthase; pfkA, phosphofructokinase; pykF, pyruvate kinase; ppc, phosphoenolpyruvate carboxylase; icd, isocitrate dehydrogenase. Reactions (BiGG identifiers): ACKr, acetate kinase; PPCK, phosphoenolpyruvate carboxykinase; PPS, phosphoenolpyruvate synthase; FBP, fructose-bisphosphatase; ICL, isocitrate lyase; MALS, malate synthase; PFK, phosphofructokinase; PYK, pyruvate kinase; PPC, phosphoenolpyruvate carboxylase; ICDHyr, isocitrate dehydrogenase (NADP).

A single GEM was used to model the average E. coli metabolic state, and we compared the simulation results with the original data from Varma and Palsson (18) (see Fig. S1 in the supplemental material). The parameters for the simulations are reported in Table 1 and 2, and the only flux constraints that we calibrated to the data were the oxygen uptake rate and the maximal acetate secretion rate. A fixed cell death rate (Table 1) was introduced as previously described, using a value from the literature (19). In these simulations, a lower absolute level of flux variation at each simulation time step was observed with the MOMA implementation (Fig. S2). We used the same GEM to reproduce the results from Enjalbert et al. (22), changing only the initial values for biomass, glucose, and acetate (Fig. 1; see also Fig. S5). Although the pFBA simulation (Fig. 1a) showed a brief shift to growth on acetate at the time of glucose exhaustion (GE) (∼4 h), the MOMA simulation predicted complete growth arrest already occurring at that point, with a minimal acetate consumption to satisfy the ATP maintenance requirement implemented in the GEM (Fig. 1b). Both simulations well captured glucose consumption and acetate secretion, but neither was able to reproduce the slow acetate consumption observed experimentally. Even after fine-tuning the constraint on acetate uptake to achieve a perfect match of the acetate consumption data from Varma and Palsson (18), the model could not reproduce the acetate concentration dynamics of the corresponding data from Enjalbert et al. (22) (data not shown). Therefore, we decided to avoid fine-tuning of the acetate uptake (Table 1). Both the pFBA and MOMA simulations showed an abrupt change in the flux distribution upon shifting from glucose consumption to acetate consumption (Fig. S3). We evaluated the agreement between the experiment and the simulation with the *R*^2^ distance between *in vivo* and *in silico* data for biomass (pFBA *R*^2^ = 0.989; MOMA *R*^2^ = 0.982), glucose (pFBA *R*^2^ = 0.993; MOMA *R*^2^ = 0.993), and acetate (pFBA *R*^2^ = 0.277; MOMA *R*^2^ = 0.409). In Fig. 2, we compare the flux distributions of our simulation results to the experimental results reported by Enjalbert et al. (22) for overexpression/underexpression of key genes associated with glucose and acetate metabolism (represented graphically in the top panels). First, we computed the flux solutions for E. coli growing on either glucose or acetate exponentially (data not shown) and compared the fluxes through the relevant reactions in E. coli growing on acetate to those in E. coli growing on glucose. Fig. 2a shows the absolute values for the flux results in the two simulations, normalized to values between 0 and 1 for direct comparison with the qualitative representation of the gene expression data (with a value of 0 for nonexpressed genes and a value of 1 for expressed genes). The simulation results were consistent with the results of the experiments, with active reactions (dark green) related to acetate consumption and anabolism (ACKr, PPCK, FBP, ICL, MALS) and inactive reactions (white) related to glycolysis (PFK and PYK) during growth on acetate and vice versa during growth on glucose. PPS did not carry flux in either simulation. We then used the simulation results presented in Fig. 1 to compare the metabolic fluxes before and after glucose exhaustion (GE), i.e., before and after the single E. coli model shifted from growth on glucose to growth on acetate. Enjalbert et al. (22) compared gene expression levels between samples taken at time GE plus 30 min and at time GE minus 115 min. However, Fig. 1 shows that according to the simulation, growth had already stopped after 30 min from the GE point. Indeed, comparing the absolute values of fluxes taken at time GE plus 30 min and at time GE minus 115 min, we found that both the pFBA and MOMA simulations qualitatively captured the downregulation trends, whereas neither simulation reproduced the observed upregulation (data not shown). Fig. 2b shows the difference in absolute values of fluxes taken at time GE plus 18 min and at time GE minus 115 min, time points where growth is still observed in pFBA simulations. In this case, both simulations qualitatively captured most of the upregulation/downregulation trends. Figure S4 shows the metabolic network (modified from the map for the E. coli core model constructed by the use of Escher [28]), with the data from the reactions performed as described for Fig. 2b highlighted and color-coded according to the gene expression data. Finally, we reproduced the other experimental scenarios from Enjalbert et al. (22) with the uniform population model, adjusting only the initial values of biomass, glucose, and acetate. We observed that a uniform shift was able to reproduce the biomass profile well when high acetate concentrations were present in the medium (Fig. S5c), while this was not the case when only low acetate concentrations were available (Fig. S5b).

**TABLE 1:** Fixed parameters for all simulations^*a*^

Parameter	Value
L.B. EX_O2 (mmol/gDW/h)	−11.5
U.B. EX_Ac (mmol/gDW/h)	3
δ (h^–1^)	0.03
$V_{\text{M}}^{\text{Glc}}$ (mmol/gDW/h)	10
$K_{\text{M}}^{\text{Glc}}$ (mM)	0.01
$V_{\text{M}}^{\text{Ac}}$ (mmol/gDW/h)	10
$K_{\text{M}}^{\text{Ac}}$ (mM)	0.01

a The lower bound (L.B.) for oxygen exchange (EX_O2) as well as the upper bound (U.B.) for acetate exchange (EX_Ac) were calibrated on the basis of data from Varma and Palsson (18). The death rate (δ) was computed assuming a cell death of 1% per generation (45) and a generation time of 20 min. The Michaelis-Menten parameters for substrate uptake are taken from a report by Gosset (46). Those parameters were also used in previously published dFBA implementations (47). gDW, grams dry weight.

**TABLE 2:** Parameters of the simulations^*a*^

Figure reference(s) or condition	Value(s)													
	BM(0) (10^−3^ g DW)	% EC_Gl_(0)	Glc(0) (mmol)	Ac(0) (mmol)	ξ (mmol /h)	ζ (mmol/h)	*t_x_* (h)	ψ_0_ (h^–1^)	$V_{\text{M}}^{\psi }$ (h^–1^)	$K_{\text{M}}^{\psi }$ (mM)	ϕ_0_ (h^–1^)	$V_{\text{M}}^{\phi }$ (h^–1^)	$K_{\text{M}}^{\phi }$ (mM)	ε
Fig. S1a and c	0.3	NA	10.8	0.4	0.0	0.0	0.0	0.0	0.0	0.0	0.0	0.0	0.0	0.0
Fig. S1b and d	0.24	NA	0.82	0.1	1.1	0.0	0.0	0.0	0.0	0.0	0.0	0.0	0.0	0.0
Fig. 1a and b, Fig. S5a	2.7	NA	15.0	0.0	0.0	0.0	0.0	0.0	0.0	0.0	0.0	0.0	0.0	0.0
Fig. 3a	2.7	0.95	15.0	0.0	0.0	0.0	0.0	0.0	0.0	0.0	0.0	0.0	0.0	0.0
Fig. 3b, Fig. S5d	2.7	0.95	15.0	0.0	0.0	0.0	0.0	0.04	0.0	0.0	0.04	0.0	0.0	0.9
Fig. S5g	2.7	0.95	15.0	0.0	0.0	0.0	0.0	0.04	0.2	30.0	0.04	0.2	5.0	0.9
Fig. S5b	3.8	NA	15.0	0.0	0.0	9.1	4.0	0.0	0.0	0.0	0.0	0.0	0.0	0.0
Fig. S5e	3.8	0.75	15.0	0.0	0.0	9.1	4.0	0.04	0.0	0.0	0.04	0.0	0.0	0.9
Fig. 4a, Fig. S5h	3.8	0.75	15.0	0.0	0.0	9.1	4.0	0.04	0.2	30.0	0.04	0.2	5.0	0.9
Fig. S5c	6.0	NA	15.0	32.0	0.0	9.1	4.0	0.0	0.0	0.0	0.0	0.0	0.0	0.0
Fig. S5f	6.0	0.75	15.0	32.0	0.0	9.1	4.0	0.04	0.0	0.0	0.04	0.0	0.0	0.9
Fig. 4b, Fig. S5i	6.0	0.75	15.0	32.0	0.0	9.1	4.0	0.04	0.2	30.0	0.04	0.2	5.0	0.9
M9G (m.c.)	2.7	0.95	15.0	0.0	0.0	0.0	0.0	0.04	0.2	30.0	0.04	0.2	5.0	0.9
M9GA (m.c.)	6.0	0.75	15.0	32.0	0.0	0.0	0.0	0.04	0.2	30.0	0.04	0.2	5.0	0.9

a NA, not in the model; BM(0), initial biomass quantity; % EC_Gl_(0), initial percentage of glucose-consumer population; Glc(0), initial glucose concentration; Ac(0), initial acetate concentration; *t_x_*, time of glucose exhaustion; m.c., mother culture. All other parameters are defined in the text.

10.1128/mSystems.00230-18.2 FIG S1 Diauxic growth of *E. coli* modeled as a uniform population in batch (a and e) and fedbatch (b and f) conditions. Simulation (connected dots; each dot represents a simulation step) data are compared to data (squares) from Varma and Palsson (18) as a function of time. Biomass (blue), glucose (red), and acetate (cyan) data are shown. The flux distribution at each time step was obtained with pFBA (a and b) or MOMA (e and f). Below each plot, left to right, are reported data corresponding to agreement between the biomass, glucose, and acetate experimental data with simulation results. Download FIG S1, PDF file, 0.4 MB. Copyright © 2019 Succurro et al. 2019 Succurro et al. This content is distributed under the terms of the Creative Commons Attribution 4.0 International license.

10.1128/mSystems.00230-18.3 FIG S2 (Left plots) Euclidean distance between the flux solution vectors at simulation steps i and i + 1 plotted against simulation time (lower *x* axis) and simulation steps (upper *x* axis, in red). (Right plots) Absolute distance between the flux data at simulation steps i and i + 1 for the reactions yielding the higher change, plotted against simulation time (lower *x* axis) and simulation steps (upper *x* axis, in red). Notice the nonlinear relation between simulation time and simulation step data, originating from the variable integrator time step choice. Download FIG S2, PDF file, 0.3 MB. Copyright © 2019 Succurro et al. 2019 Succurro et al. This content is distributed under the terms of the Creative Commons Attribution 4.0 International license.

10.1128/mSystems.00230-18.4 FIG S3 (Left plots) Euclidean distance between the flux solution vectors at simulation steps i and i + 1 plotted against simulation time (lower *x* axis) and simulation steps (upper *x* axis, in red). (Right plots) Absolute distance between the flux data at simulation steps i and i + 1 for the reactions yielding the higher change, plotted against simulation time (lower *x* axis) and simulation steps (upper *x* axis, in red). Notice the nonlinear relation between simulation time and simulation step data, originating from the variable integrator time step choice. Download FIG S3, PDF file, 0.1 MB. Copyright © 2019 Succurro et al. 2019 Succurro et al. This content is distributed under the terms of the Creative Commons Attribution 4.0 International license.

10.1128/mSystems.00230-18.5 FIG S4 Escher (28) map of the metabolic network of the E. coli core. The pathways highlighted in different colors are those that were found to differ between the time before and the time after glucose exhaustion (GE at −115 min and GE at +18 min, respectively) in the simulations. In red are highlighted the reactions found to carry higher fluxes after the switch to growth on acetate, in blue those carrying lower fluxes, and in gray those that did not change. The names of the reactions are color-coded in the same way but in accordance with the experimental results from Enjalbert et al. (22). Download FIG S4, EPS file, 2.2 MB. Copyright © 2019 Succurro et al. 2019 Succurro et al. This content is distributed under the terms of the Creative Commons Attribution 4.0 International license.

10.1128/mSystems.00230-18.6 FIG S5 Simulations of the three experiments from Enjalbert et al. (22). (Left column) Growth on 15 mM glucose. (Middle column) Growth on 15 mM glucose and fed-batch 4 mM acetate. (Right column) Growth on 15 mM glucose and fed-batch 32 mM acetate. (Top row) Uniform population model. (Middle row) Subpopulation model with stochastic shift (fixed-rate transition). (Bottom row) Subpopulation model with responsive shift (Hill function transition). Download FIG S5, PDF file, 0.3 MB. Copyright © 2019 Succurro et al. 2019 Succurro et al. This content is distributed under the terms of the Creative Commons Attribution 4.0 International license.

### E. coli diauxie modeled with a mixed population.

**FIG 3:**
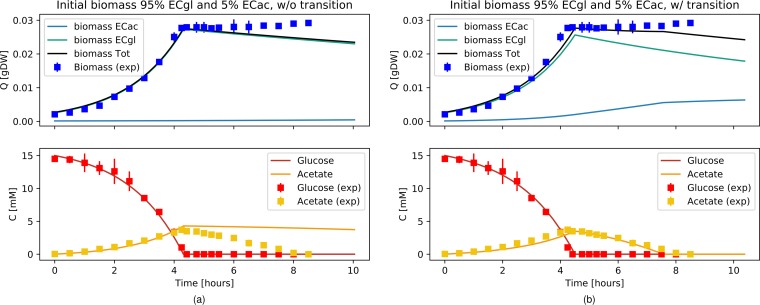
Diauxic growth of E. coli modeled as a mixture of two E. coli populations, EC_Gl_ and EC_Ac_, growing exclusively on glucose and acetate, respectively, without (a) or with (b) the possibility of shifting from one population to the other. Simulation data (lines) are compared to data from Enjalbert et al. (22) (squares) as a function of time. The upper plots show simulation results (obtained using pFBA) for EC_Gl_ and EC_Ac_ biomass data (light blue and aqua, respectively) and the observable E. coli biomass data (black line simulation, blue dots). The bottom plots show glucose and acetate (red and yellow, respectively).

**FIG 4:**
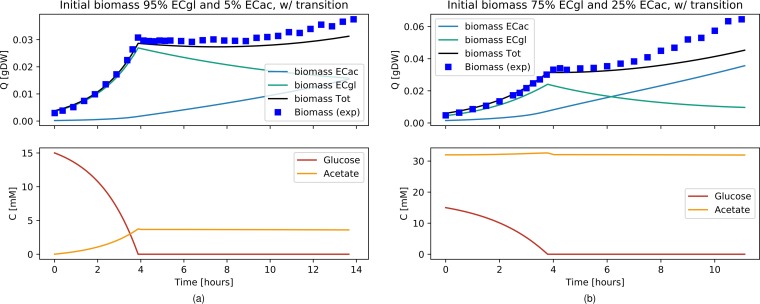
Diauxic growth of E. coli modeled as a mixture of two E. coli populations, EC_Gl_ and EC_Ac_, growing exclusively on glucose and acetate, respectively, with the possibility of shifting from one population to the other. (a) E. coli grows on 15 mM glucose; after the glucose was exhausted, the acetate concentration was kept at about 4 mM. (b) E. coli grows on 15 mM glucose and 32 mM acetate; after the glucose was exhausted, the acetate concentration was maintained at around the same concentration. The upper plots show simulation results (obtained using pFBA) for EC_Gl_ and EC_Ac_ biomass data (light blue and aqua lines, respectively) and the observable E. coli biomass data (black line simulation, blue dots) (data from Enjalbert et al. [22]). The bottom plots show simulation results for glucose and acetate (red and yellow lines, respectively).

We used two GEMs (and the same parameter values as before) to model E. coli monocultures as a mixture of two populations, one adapted to grow on glucose and one adapted to grow on acetate. The two models, the E. coli glucose (EC_Gl_) model and the E. coli acetate (EC_Ac_) model, were hence constrained to exclusively take up the corresponding carbon source. Two transition functions, dependent on acetate or glucose concentrations, were introduced to model cellular differentiation and cellular shift from one population to the other (see Materials and Methods for details). We ran simulations to compare the different scenarios investigated experimentally by Enjalbert et al. (22). The initial values for biomass, glucose, and acetate were adjusted to the corresponding data sets. The transition rates, as well as the initial population ratios, were chosen following the assumption, supported by a simple mathematical model, that populations in constant environments will converge to a constant ratio (see Text S1 in the supplemental material for details). Data in Fig. 3 show simulations performed under the same conditions as those described for Fig. 1a, with the same absolute initial biomass values, distributed in this case as 95% EC_Gl_ and 5% EC_Ac_. This initial ratio was chosen by considering the range of steady-state values for the population ratio (reported in Table S1 in the supplemental material) as well as considering that it is reasonable to assume that a higher number of cells would be adapted to grow on glucose, which is the carbon source on which laboratory cultures are usually maintained. Data in Fig. 3a show the simulation results for a scenario without transitions between the two states, whereas the results of Fig. 3b were obtained with active transition functions, defined here by constant transition rates as reported in Table 2. Although both panels a and b of Fig. 3 capture well the biomass (*R*^2^ = 0.987 and *R*^2^ = 0.990, respectively) and glucose concentrations (*R*^2^ = 0.996 and *R*^2^ = 0.997, respectively), only the simulation that included the population transition realistically reproduced the acetate consumption levels (*R*^2^ = 0.336 and *R*^2^ = 0.951 respectively) as well as a lag phase before culture crash. Neither of the simulations captured the eventual recovery of growth hinted at by the last data points. We reproduced two other results (where only biomass measurements were available) from Enjalbert et al. (22), again using the same GEMs and changing only the initial conditions (biomass quantity and distribution among EC_Gl_ and EC_Ac_) and the experimental setup accordingly. By modeling the population transition with the same constant rate, we were able to explain the biomass profile in the case where E. coli was grown on 15 mM glucose and, after glucose exhaustion, the acetate concentration was maintained at around 4 mM (Fig. S5e) (*R*^2^ = 0.986), but not in the case where E. coli was grown on 15 mM glucose and 32 mM acetate and, after glucose exhaustion, the acetate concentration was maintained at the same high level (Fig. S5f) (*R*^2^ = 0.727). We therefore introduced a dependency of the transition functions on the substrate concentration (see Materials and Methods for details) that well captures all the experimental scenarios with a unique set of parameters (Fig. S5g, h, and i). Data in Fig. 4a show that an E. coli population starting with 95% EC_Gl_ and 5% EC_Ac_ describes well the biomass dynamics (*R*^2^ = 0.985) and matches the glucose exhaustion point, observed after around 4 h when acetate was maintained at 4 mM. Again, without fine-tuning the GEM simulation parameters, Fig. 4b shows that an E. coli population starting with 75% EC_Gl_ and 25% EC_Ac_ reproduced the biomass measurements (*R*^2^ = 0.940) and the glucose exhaustion point after around 4 h also in the experimental setup with acetate maintained at 32 mM. The effect of adjusting the initial biomass ratios in the different experimental conditions is shown in Fig. S6. Overall, the simulations starting with 95% EC_Gl_ and 5% EC_Ac_ or 75% EC_Gl_ and 25% EC_Ac_ did not show strong differences, but further reducing the percentage of EC_Gl_ (and leaving the range of steady-state values of Table S1) resulted in drastic changes to the shape of the growth curves. The initial condition of a 75% EC_Gl_ and 25% EC_Ac_ population distribution for Fig. 4b is also justified by a difference in the initial experimental values for the biomass quantity (see Fig. S7).

10.1128/mSystems.00230-18.1 TEXT S1 Simple mathematical model of two coexisting subpopulations in constant environments. Download Text S1, PDF file, 0.1 MB. Copyright © 2019 Succurro et al. 2019 Succurro et al. This content is distributed under the terms of the Creative Commons Attribution 4.0 International license.

10.1128/mSystems.00230-18.7 FIG S6 Simulation of two subpopulations (EC_Glc_ and EC_Ac_) growing on glucose only (a) or on glucose and (b) a low level of acetate or (c) a high level of acetate. Experimental data from Enjalbert et al. (22) are shown as blue circles. The different curves show simulation results with same parameters except for initial biomass ratios. Download FIG S6, PDF file, 0.2 MB. Copyright © 2019 Succurro et al. 2019 Succurro et al. This content is distributed under the terms of the Creative Commons Attribution 4.0 International license.

10.1128/mSystems.00230-18.8 FIG S7 Results from simulating EC_Glc_ and EC_Ac_ growing on glucose and a high level of acetate (see main text). The initial biomass value was taken from time point “GE—4hr” (a) or “GE—5hr” (b) in the data from Enjalbert et al. (22), with the initial population ratio as reported on the plot. All other simulation parameters were the same and were as described in the manuscript. Download FIG S7, PDF file, 0.07 MB. Copyright © 2019 Succurro et al. 2019 Succurro et al. This content is distributed under the terms of the Creative Commons Attribution 4.0 International license.

10.1128/mSystems.00230-18.10 TABLE S1 Scan of the allowed values for ϕ and ψ under different conditions and respective values of log Γ. Under low-acetate conditions (minimal ψ), any value of ϕ is allowed, but we report a subset here. Download Table S1, PDF file, 0.02 MB. Copyright © 2019 Succurro et al. 2019 Succurro et al. This content is distributed under the terms of the Creative Commons Attribution 4.0 International license.

### Lag time for growth on acetate explained with population distribution.

**FIG 5:**
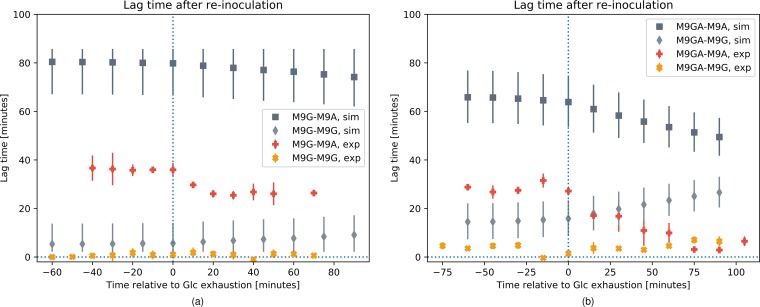
Simulation data (dark and light gray points) and experimental data (orange and yellow points) (data from Enjalbert et al. [22]) for the delay in the growth of daughter cultures before they reached maximal growth after the medium switch. Mother cultures are grown on either 15 mM glucose (M9G) (a) or 15 mM glucose and 32 mM acetate (M9GA) (b). Daughter cultures are reinoculated into fresh media with either 15 mM glucose (M9G; square and plus markers) or 45 mM acetate (M9A; diamond and cross markers). The simulation error bars are obtained by adjusting the initial population ratios (obtained by sampling the simulated mother cultures) by ±15%.

Enjalbert et al. (22) showed different trends in the lag times of E. coli cultures required to achieve maximal growth after GE. In their switch experiments, they sampled at different time points “mother cultures” of E. coli cells growing under batch conditions on 15 mM glucose alone (“M9G” condition) or on 15 mM glucose and 32 mM acetate (“M9GA” condition) and reinoculated the sampled cells as “daughter cultures” into fresh medium exclusively containing glucose (M9G condition) or acetate (M9A condition). We replicated this experiment *in silico* by running first simulations under the M9G and M9GA conditions. For the M9G mother culture, we used the simulation of the mixed EC_Gl_ and EC_Ac_ population shown in Fig. 3b, because the experimental conditions were the same. We did not have an experimental reference data set for the M9GA mother culture, and we simulated a new scenario similar to that shown in Fig. 4b, with the same initial population composed of 75% EC_Gl_ and 25% EC_Ac_ but without the feeding of additional acetate. The GE time points were about 4.6 h for M9G and 3.9 h for M9GA, consistent with the observations of Enjalbert et al. (22) (data not shown). The *in silico* mother cultures were sampled at regular time intervals to obtain the initial biomass distribution of EC_Gl_ and EC_Ac_ for the daughter cultures (reported in Table 3), and the lag time was computed for each daughter culture (see Materials and Methods for details). Data in Fig. 5 show the simulation results compared with the experimental data from Enjalbert et al. (22). The error bars were obtained for the simulated lag time data by adjusting the initial biomass ratio of the daughter cultures by ±15%. A quantitative agreement between simulation and experimental results was achieved only in the M9G-M9G switch experiment (Fig. 5a) with the correct prediction of almost zero lag time for the daughter cells, but the trend for the delay to reach maximal growth was in general qualitatively reproduced also for the other scenarios. According to the simulations, cultures switched from M9G to M9A (Fig. 5a) need about 1.5 h before reaching maximal growth, which is more than twice the duration observed experimentally. For cultures pregrown in M9GA (Fig. 5b), we observed both in simulations and in experiments a decreasing lag time for daughter cultures sampled after GE for the M9GA-M9A switch and an increasing lag time for the M9GA-M9G switch. Additional studies are represented in Fig. S8. In particular, panels a to d of Fig. S8 show the dependence of the lag time in the daughter cultures on the maximal transition values and panels e to h of Fig. S8 show the same dependence, including the distribution of the biomass ratio in the mother cultures, for a limited set of parameters.

**TABLE 3:** Percentage of EC_Gl_ biomass under M9G and M9GA conditions at indicated time points relative to glucose exhaustion

Condition	% EC_Gl_ biomass at time (h):										
	−1.0	−0.75	−0.5	−0.25	0	0.25	0.5	0.75	1.0	1.25	1.5
M9G	95.1	95.0	94.8	94.5	94.3	93.2	92.3	91.4	90.6	89.4	88.2
M9GA	78.7	78.6	78.1	77.3	76.5	73.2	70.1	67.2	64.5	62.0	59.7

10.1128/mSystems.00230-18.9FIG S8(a to d) Phase space of the lag time for typical daughter cultures from M9G mothers (a and b) and M9GA mothers (c and d) grown under M9G (a and c) or in M9A (b and d) conditions. When the glucose (acetate) value is zero, the transition ϕ (ψ) is in principle reduced to the noise component ϕ_0_ (ψ)_0_. (e to h) Effects of adjusting the transition parameters $V_{M}^{\psi }$ and $V_{M}^{\phi }$ to values of 0.2, 0.5, and 0.8 and *K*^ψ^ to values of 10 (e and f) and 30 (g and h) for the population ratio (top panel in the plots) and for the lag time (bottom panel in the plots) for daughter cultures sampled at different times with respect to the time of glucose exhaustion. Each point in panels e and g represents results from a daughter culture from the M9G mother culture, while each point in panels f and h represents results from the M9GA mother culture. Download FIG S8, PDF file, 0.2 MB.Copyright © 2019 Succurro et al.2019Succurro et al.This content is distributed under the terms of the Creative Commons Attribution 4.0 International license.

## DISCUSSION

We have investigated a fundamental example of metabolic adaptation, namely, the diauxic growth of E. coli on glucose and acetate, aiming to test whether a dynamic metabolic modeling approach can capture diauxie in monocultures of E. coli as the observable emergent result of individual (subpopulation) behavior. To this end, we first developed a modeling framework to integrate dynamic models (ODE systems) with structural models (metabolic networks) and then performed simulations to reproduce published experimental results *in silico*.

### Avoiding fine-tuning of model parameters.

One recurrent criticism of stoichiometric and constraint-based modeling approaches, such as FBA, is that they can easily be adjusted to reproduce experimental results by *ad hoc* changes of flux constraints. Indeed, we observed that a condition-specific fine-tuning of the constraint on acetate uptake could reproduce fairly well the growth dynamics of the different experiments (data not shown). However, the change of such a constraint from one experimental condition to another is not biologically justified. Although some extensions of the FBA approach such as FBA with molecular crowding (FBAwMC [29]) provide reasonable ways to constrain the metabolic fluxes and were shown to reproduce carbon consumption hierarchies, they also require extensive parameterization. We therefore chose to use the basic FBA approach, limiting the number of constraints imposed and with parameters mostly from experimental measurements (Table 1). In the case of oxygen uptake and acetate secretion, we calibrated the constraints using data from Varma and Palsson (18), where an E. coli diauxic shift from glucose to acetate was first simulated using a genome-scale model. The FBA parameters were left unchanged to reproduce the independent experiments reported by Enjalbert et al. (22). The use of an independent set of data to calibrate the FBA model parameters is a possible way to improve the confidence in subsequent results. Further model parameters of the ODE system were chosen according to reasonable hypotheses and were adjusted slightly to achieve fair agreement with the experimental results in a manner that was consistent among all the simulations. The initial conditions were specific to the experiments that we aimed to reproduce.

### Standard dFBA allows for abrupt metabolic readjustments.

The flux distributions obtained from FBA solutions represent an average picture of the metabolic state of a population, which is in general modeled using a single genome-scale model. Therefore, standard dFBA implementations, in which the FBA constraints evolve according to the updated external conditions, reproduce the average change in metabolic state of the population in response to external variations. This is equivalent to assuming that a population undergoes a coordinated, uniform metabolic shift under changing environmental conditions. Furthermore, such transitions are generally abrupt with dFBA models. We therefore tested two alternative approaches to simulate the diauxic shift in uniform E. coli monocultures, solving the FBA problems either with pFBA (mostly equivalent to the usual dFBA implementations) or with an adaptation of the MOMA algorithm. In the latter case, instead of minimizing the difference in flux distribution between a “wild-type” GEM and a modified one (original MOMA implementation), we used the same concept to integrate the dFBA system while also imposing the following condition: at initial time *t_i_*, the flux solution differs minimally from that at time *t_i_*_-1_, where the time steps are set by the integration routine. In contrast to our expectations, however, this approach did not achieve smoother metabolic adjustments in the system in response to the changing external conditions. Instead, both implementations resulted in abrupt changes in the flux distributions following the shift from glucose to acetate metabolism (see Fig. S3 in the supplemental material). More-sophisticated implementations of a dynamic MOMA model (e.g., computing the minimal adjustment based on a subset of biologically relevant variables) might succeed in achieving smooth metabolic transitions but would require the introduction of additional parameters and *ad hoc* biological hypotheses. In a similar way, biologically justified extensions of FBA such as FBAwMC (29) might provide better descriptions of an average and uniform population-level metabolism but typically need the empirical determination of large numbers of organism-specific parameters.

### Monocultures can be modeled as multisubpopulation systems to capture individual heterogeneity.

With the introduction of two basic assumptions (first, that there are two distinct metabolic states consuming either glucose or acetate; second, that transition from one state to the other is driven by glucose and acetate concentrations), we were able to capture all the experimental trends published by Enjalbert et al. (22) with the same computational model. The transitions between the two states were modeled as Hill functions of the corresponding substrate concentrations with a noise offset representing a constant, small noise component in cell regulation. Although other transition laws could have been chosen, Hill functions conveniently model concentration-dependent shifts between two states. For example, when acetate is highly abundant, more cells in the glucose consumption state shift to the acetate consumption state in response to the change in environment. Finally, the introduction of a transition efficiency term was motivated by the observation that cells can get “lost in transition,” an effect that was estimated to account for the death of ∼7% of yeast cells, which cannot initiate glycolysis following a shift to high glucose levels (30). Using a simple mathematical model (see Text S1 in the supplemental material), we identified ranges for the parameters of the transition functions and selected reasonable values that would return good agreement between simulations and experiments. Both the values for the constant transition rate (4% h^–1^) and the values for the maximal transition rate (20% h^–1^) were in good agreement with measured average protein turnover rates in E. coli cultures from the literature (31–33). Simulation results were mostly in very good agreement with the experimental data, and our results strongly further support the idea, suggested over the last few years by results from independent studies of different organisms (21, 25, 34), that monocultures represent an ensemble of subpopulations in different metabolic states that are partially regulated by the environmental conditions. When the acetate concentrations were too low to support growth, it was sufficient to model the transition as a constant random process. In contrast, in order to reproduce the data under conditions with high acetate concentrations, we needed to introduce an active transition rate dependent on substrate concentrations. Interestingly, this assumption alone was sufficient to model the experimentally observed growth rate, without further fine-tuning of model parameters. The introduction of substrate-dependent transition functions is also consistent with the experimental observations of Kotte et al. (27), supporting the hypothesis that a monoculture undergoes diversification in response to environmental changes.

### The lag phase corresponding to growth on different substrates can be explained by population distributions.

With standard dFBA simulations, the metabolic transition during the shift from one carbon source to another is abrupt, and no lag phase is observable. Such an outcome is rarely the case, and, most remarkably, the duration of the lag phase between the exhaustion of the favored carbon source and the resumption of optimal growth on the alternate carbon source is highly variable under different environmental conditions. This observation can easily be explained as an emergent property of subpopulation dynamics. Our simulations are consistent with the explanation that the delay in the resumption of full growth actually depends on the relative abundances of the two subpopulations. Although the simulation results did not reproduce the experimental data quantitatively, all qualitative trends were fully explained. Several factors may explain these discrepancies. For example, the lack of experimental data concerning the mother cultures (in terms of biomass, glucose, and acetate dynamics) made it impossible to calibrate the initial model population. This could introduce a significant bias in the later sampling and determination of the subpopulation ratio, thus strongly influencing the quantification of the lag time, which is highly correlated with the population distribution (Fig. S8). Solopova et al. (25) showed that the density of a Lactococcus lactis population (translating in practice to the rate at which the primary carbon source was consumed) played a significant role in determining the proportion of cells successfully transitioning to growth on the secondary carbon source. The connection between lag time and subpopulation distribution could in principle be exploited to estimate initial population distributions from lag time measurements. However, it is difficult to assess the robustness and reliability of such predictions with the currently available data, and further investigation, including experiments devoted to determination of initial conditions, is therefore required. An additional source of the discrepancies between our quantitative results and the experimental measurements could have been the experimental procedure itself. For example, abrupt changes in conditions, such as the reinoculation of daughter cultures into a different medium in the switch experiments, might select for additional adaptation strategies. Interestingly, we observed a dramatic improvement in the quantitative agreement between experiment and simulation by relaxing the condition imposing no growth for populations inoculated on the “wrong” carbon source (data not shown). By allowing the glucose-consuming population sampled from glucose mother cultures to growth more slowly on acetate, we mimicked a situation in which cells store resources and are able to survive a bit longer. On the other hand, allowing reduced growth on acetate (glucose) for the glucose consumer (acetate consumer) population that was exposed to both carbon sources in the mixed mother cultures could be a proxy for a memory effect. Bacterial cells do show memory effects in response to changes in environmental conditions (26), but more-systematic experiments would be necessary to carefully and reproducibly determine the lag times as functions of external parameters to explore this potential explanation further. Finally, data obtained using a recent stochastic model of the regulatory network of diauxic growth in E. coli suggest that the limitations of biological sensors are responsible for the lag phase (35). From these results, we can infer that the transition functions, which currently depend on the absolute concentration of one carbon source at a time, might not be able to capture the fine details of population shifts in our model. A possible extension would be to introduce more-complex transition mechanisms dependent on the relative concentrations of primary and secondary carbon sources, a process whose elucidation would need dedicated experiments for the construction and validation of the new transition functions.

### Subpopulations in the dynamic metabolic modeling approach.

We developed a modeling framework to perform FBA simulations using embedment in a system of ODEs. Building on previous methods and approaches (19, 36), we further extended the standard dFBA implementation and introduced novel concepts. In particular, standard dFBA approaches assume that fluxes can instantaneously change to adapt to new environmental conditions, and flux solutions at subsequent time steps might differ significantly. This is an obvious limitation when aiming to capture diauxic shift, where lag phases, highly dependent on the environmental conditions, are typically observed. We implemented the MOMA algorithm (originally developed to model the response to genetic perturbations in static FBA) in dFBA to minimize the metabolic adjustments between different time points. Furthermore, we integrated dynamic mechanisms into dFBA that cannot be included in metabolic models, such as population transitions. Indeed, although the use of dFBA to model subpopulations bears some similarities to the use of other platforms for the simulation of microbial communities, a notable difference in our formulation is the capacity of subpopulations to interconvert. The current study relied on the *a priori* knowledge that only two carbon sources would be available to E. coli, thus motivating the development of a two-subpopulation community, but in principle, an arbitrary number of subpopulations can be defined and more generic transition functions introduced. Further experiments, in particular, single-cell studies, could be designed to define and parameterize these transition functions. Thanks to the object-oriented (OO) design of the framework, it is relatively easy to introduce other functions regulating the constraints on specific reaction fluxes in the FBA problem. In this way, different hypotheses can be extensively tested to improve understanding of how to capture regulatory dynamics in dFBA. Notably, the methods developed in this framework to study population heterogeneity could then be transferred to other platforms that are more specific for microbial community modeling where different features are implemented (e.g., spatial structure [19] or community-level objectives [37]). Finally, the framework could also be developed further to include stochastic mechanisms, such as mutations that would alter the function of metabolic genes. Indeed, our implementation of the dFBA algorithm is able to call different methods at each time step, e.g., to update the flux rates, and a regulatory function with random components could in principle be defined.

### Outlook.

There is extensive experimental evidence that bacteria differentiate into subpopulations as a result of survival strategies (25, 27). Simulations based on standard dFBA model the dynamics of cells by predicting the putative average behavior of a whole population. For example, if a population of cells globally utilizes a combination of two carbon sources, dFBA would predict metabolic states in which the two carbon sources are utilized simultaneously. Our model assumes that cells are either in the glucose-consuming state or the acetate-consuming state, with an instantaneous transition between these two subpopulations that follows a simplistic rule which cannot capture intermediate states. This simplification is both practical and plausible for observing population dynamics as the emergent properties of individual behavior, and it works well in dynamically changing environments with a continuous transition. However, rather than having a well-defined metabolic state, especially during the transition between states, cells might exhibit a mixed state, which could be described as a superposition of “pure” states, analogous to the state vectors in quantum physics. Furthermore, our approach suggests a fundamental difference in the strategies used to account for metabolic fluxes in heterogeneous populations, because the average fluxes in a uniform population might differ from the cumulative average fluxes of subpopulations. Further investigations of this novel concept of superimposed metabolic states will provide a promising new approach to study the principles of metabolic regulation.

## MATERIALS AND METHODS

### FBA methods.

In stoichiometric models, the stoichiometric matrix **S**(*m*×*n*) is defined with the row and column dimensions corresponding to the numbers of metabolites *m* and reactions *n* respectively, the elements *s_ij_* being the stoichiometric coefficients of metabolite *i* taking part in reaction *j*. FBA defines and solves the following LP problem:

(1) $$\text{maximize}\;\overset{⃗}{z}$$

subject to:

(2) $$\;S\overset{⃗}{v}=0$$

(3) $$\text{ l.b}._{j}\leq v_{j}\leq \text{u.b}._{j}$$

with l.b._*j*_ and u.b._*j*_ representing, respectively, a lower and upper bound on flux *v_j_*.

The steady-state assumption (equation 2) gives a system of equations that is underdetermined and has an infinite number of solutions. Constraints on the fluxes (equation 3) allow us to restrict the solutions to a convex solution space but still result in an infinite number of solutions. The definition of an objective (equation 1) selects one solution, but this is still generally not unique for large (genome-scale) metabolic networks.

We consider two extensions to the FBA problem definition, namely, pFBA (14) and MOMA (15). We then use these two methods to solve the FBA problem in an approach similar to dFBA (16). Assuming that metabolism evolves toward the efficient utilization of resources, pFBA finds the minimal flux distribution that returns the same objective as that defined by the FBA problem. We use the pFBA implementation from COBRApy (38) with maximal flux through the biomass reaction as the objective function. Considering that metabolism must respond quickly to perturbations, MOMA implements a quadratic algorithm to find the FBA solution after gene deletion that is most similar to the optimal wild-type configuration. In our case, we do not introduce modifications to the metabolic network but rather require that the MOMA solution obtained at time *t_i_*_-1_ is used to compute the MOMA solution at time *t_i_* as the minimally different solution that satisfies the objective function. Also in this case, the objective function is maximal flux through the biomass reaction. We use the MOMA implementation from COBRApy (38) in the linear approximation, with a slight modification to allow the LP problem to be reset in an iterative manner, which is necessary to run MOMA within the dFBA approach.

### Modeling framework integrating ODE and FBA.

In the SOA of dFBA, the boundary conditions in equation 3 are updated at discrete time steps according to the solution of the FBA problem in the previous time interval. Assuming quasi-steady-state conditions, i.e., that metabolism readjustments are faster than external environmental changes, dFBA can approximate the dynamic response of a GEM to a changing environment. Our approach is an extension of dFBA. The model is built as a system of ODEs, whose dimension depends on the dynamics to be modeled. Each ODE describes the variation in time of biomass, metabolites, or other regulatory/dynamic processes. The biomasses and the metabolites can be but are not necessarily linked to the corresponding variables in a GEM. Their ODEs vary according to a function that can then depend on the flux solutions $\overset{⃗}{v}$ as follows:

(4) $$\frac{dq_{i}}{dt}=F(\overset{⃗}{p};\overset{⃗}{v},\overset{⃗}{q},t)$$

The ODE system is then solved using integration routines with an automated choice of time step. Each integration step solves the FBA problem (or pFBA or MOMA problem) to obtain the current reaction rates for equation 4, updates the metabolite quantities according to the FBA solution, recomputes the flux boundaries of equation 3 according to specific reaction kinetics (typically Michaelis-Menten enzyme kinetics), and redefines the FBA problems with the new boundaries and/or other regulatory mechanisms defined by the user.

The modeling framework is written in Python (Python Software Foundation, https://www.python.org/) following the object-oriented programming (OOP) paradigm for efficiency and flexibility. The framework uses functionality from the following third-party packages: numpy (39), scipy (40), matplotlib (41), COBRApy (38), and pandas (42). In particular, we use COBRApy methods to solve the FBA problems and Python integrators from the scipy.integrate method ode to solve the system of ODEs.

### E. coli uniform population model.

We used a previously reported core version of E. coli GEM (43) downloaded from http://bigg.ucsd.edu/models/e_coli_core. The E. coli EC_any_ model is constrained with respect to the consumption of “any” carbon source (i.e., glucose [Gl] and acetate [Ac]) solely by the environmental conditions, and the lower bound of the exchange reactions (EX_Gl_e and EX_Ac_e, respectively) follows two simple Michaelis-Menten kinetics equations:

(5) $$\text{l.b}._{\text{EX\_Gl\_e}}^{{\text{EC}}_{\text{any}}}=-V_{M}^{\text{Gl}}\frac{\left[\text{Gl}\right]}{[\text{Gl}]+K_{M}^{\text{Gl}}}$$

(6) $$\text{l.b}._{\text{EX\_Ac\_e}}^{{\text{EC}}_{\text{any}}}=-V_{M}^{\text{Ac}}\frac{\left[\text{Ac}\right]}{[\text{Ac}]+K_{M}^{\text{Ac}}}$$

The ODE system is defined as follows:

(7) $$\frac{d{\text{BM}}_{{\text{EC}}_{\text{any}}}}{dt}=v_{\mu }^{{\text{EC}}_{\text{any}}}\cdot {\text{BM}}_{{\text{EC}}_{\text{any}}}-\delta {\text{BM}}_{{\text{EC}}_{\text{any}}}$$

(8) $$\frac{d\text{Gl}}{dt}=v_{\text{EX\_Gl\_e}}^{{\text{EC}}_{\text{any}}}\cdot {\text{BM}}_{{\text{EC}}_{\text{any}}}+\xi_{\text{fed-batch}}$$

(9) $$\frac{d\text{Ac}}{dt}=v_{\text{EX\_Ac\_e}}^{{\text{EC}}_{\text{any}}}\cdot {\text{BM}}_{{\text{EC}}_{\text{any}}}$$

where *v_μ_* is the reaction rate of the biomass function (proxy for growth rate) in the FBA model, *δ* is the cell death rate, and *ξ*_fed-batch_ is a positive rate under fed-batch conditions and zero under batch conditions. Parameters and initial conditions are summarized in Table 2. Either pFBA or MOMA can be used to solve the FBA problem.

### E. coli mixed-population model.

Two E. coli core models are loaded and defined as either a glucose consumer (EC_Gl_) model or an acetate consumer (EC_Ac_) model by switching off uptake of the other carbon source as follows:

(10) $$\mathrm{l.b}._{\text{EX\_Gl\_e}}^{{\text{EC}}_{\text{Gl}}}=-V_{M}^{\text{Gl}}\frac{\left[\text{Gl}\right]}{[\text{Gl}]+K_{M}^{\text{Gl}}}$$

(11) $$\mathrm{l.b}._{\text{EX\_Gl\_e}}^{{\text{EC}}_{\text{Ac}}}=0$$

(12) $$\mathrm{l.b}._{\text{EX\_Ac\_e}}^{{\text{EC}}_{\text{Gl}}}=0$$

(13) $$\mathrm{l.b}._{\text{EX\_Ac\_e}}^{{\text{EC}}_{\text{Ac}}}=-V_{M}^{\text{Ac}}\frac{\left[\text{Ac}\right]}{[\text{Ac}]+K_{M}^{\text{Ac}}}$$

The ODE system is defined as follows:

(14) $$\frac{d{\text{BM}}_{{\text{EC}}_{\text{Gl}}}}{dt}=(v_{\mu }^{{\text{EC}}_{\text{Gl}}}-\psi -\delta )\cdot {\text{BM}}_{{\text{EC}}_{\text{Gl}}}+\varepsilon \phi {\text{BM}}_{{\text{EC}}_{\text{Ac}}}$$

(15) $$\frac{d{\text{BM}}_{{\text{EC}}_{\text{Ac}}}}{dt}=(v_{\mu }^{{\text{EC}}_{\text{Ac}}}-\phi -\delta )\cdot {\text{BM}}_{{\text{EC}}_{\text{Ac}}}+\varepsilon \psi {\text{BM}}_{{\text{EC}}_{\text{Gl}}}$$

(16) $$\frac{d\text{Gl}}{dt}=v_{\text{EX\_Gl\_e}}^{{\text{EC}}_{\text{Gl}}}\cdot {\text{BM}}_{{\text{EC}}_{\text{Gl}}}+v_{\text{EX\_Gl\_e}}^{{\text{EC}}_{\text{Ac}}}\cdot {\text{BM}}_{{\text{EC}}_{\text{Ac}}}+\xi_{\text{fed-batch}}$$

(17) $$\frac{d\text{Ac}}{dt}=v_{\text{EX\_Ac\_e}}^{{\text{EC}}_{\text{Gl}}}\cdot {\text{BM}}_{{\text{EC}}_{\text{Gl}}}+[v_{\text{EX\_Gl\_e}}^{{\text{EC}}_{\text{Ac}}}+\zeta \cdot H(t-t_{x})]\cdot {\text{BM}}_{{\text{EC}}_{\text{Ac}}}$$

where *ζ* · *H*(*t* − *t_x_*) is a heaviside function activated at time *t_x_* of glucose exhaustion in order to keep the acetate level constant, ψ and ϕ are functions that model the cellular shift from EC_Gl_ to EC_Ac_ and from EC_Ac_ to EC_Gl_, respectively, and 0<ε<1 is a positive factor representing the transition efficiency. The *ψ* and *ϕ* functions are modeled as Hill functions with a noise offset as follows:

(18) $$\psi ([\text{Ac}])=\psi_{0}+V_{M}^{\psi }\frac{{\left[\text{Ac}\right]}^{n}}{{\left[\text{Ac}\right]}^{n}+K_{M}^{\psi }{}^{n}}$$

(19) $$\phi ([\text{Gl}])=\phi_{0}+V_{M}^{\phi }\frac{{\left[\text{Gl}\right]}^{n}}{{\left[\text{Gl}\right]}^{n}+K_{M}^{\phi }{}^{n}}$$

where they are constant transition rates for $V_{M}^{\phi }=V_{M}^{\psi }=0$. For the simulations presented here, we used a Hill coefficient value of *n *=* *5. Indeed, the simulations seemed to work best for a transition function with a high degree of cooperativity, and the results are robust with respect to small deviations relative to this value. The other parameters and initial conditions, specific to the different simulations, are summarized in Table 2. For mixed-population simulations, pFBA is used to solve the FBA problem.

### Switch experiment simulations.

Two E. coli mixed-population model simulations are run as “mother cultures” as shown in Table 2 for M9G and M9GA conditions (glucose and glucose plus acetate, respectively). From each mother culture, we sample 11 time points between –1 and +1.5 h from the corresponding GE time (4.6 h for M9G and 3.9 h for M9GA) to obtain the biomass ratio between EC_Gl_ and EC_Ac_ used as the initial condition for the reinoculation simulations. The percentage of EC_Gl_ biomass at these time points is shown in Table 3. The daughter cultures are then grown under M9G glucose-only or M9A acetate-only conditions (see Table 2), yielding 44 simplified simulations, corresponding to 11 for each of the following 4 switch experiments: M9G to M9G, M9G to M9A, M9GA to M9G, and M9GA to M9A. For each simulation, the lag time is computed according to Enjalbert et al. (22):

(20) $$t_{\text{lag}}=t_{1}-\frac{\ln(X_{1}/X_{0})}{\mu_{\text{max}}}$$

where *X*_0_ is the total initial E. coli biomass, *X*_1_ is the total E. coli biomass value at time *t*_1_ (1.5 h as described previously [22]), and *μ*_max_ values are used as described by Enjalbert et al. (22).

### Published experimental data.

Experimental data (values with standard deviations, when available) from Enjalbert et al. (22) were kindly provided by B. Enjalbert. The data from Varma and Palsson (18) were extracted from the original publication using WebPlotDigitizer (44).

### Data availability.

The version of the modeling framework used to obtain the results presented in this manuscript (v1.1) is publicly available with instructions to install and run simulations at https://github.com/QTB-HHU/daphne_ecoli-diauxie. The development version is hosted on https://gitlab.com/asuccurro/dfba-ode-framework, and people interested in contributing can request access by contacting A. Succurro (corresponding author).
