# Supplementary figures and images for: Emergent Subpopulation Behavior Uncovered with a Community Dynamic Metabolic Model of Escherichia coli Diauxic Growth
Source: mSystems. 2019 Jan 15;4(1):e00230-18. doi: 10.1128/mSystems.00230-18 (PMC6446979; doi:10.1128/mSystems.00230-18)

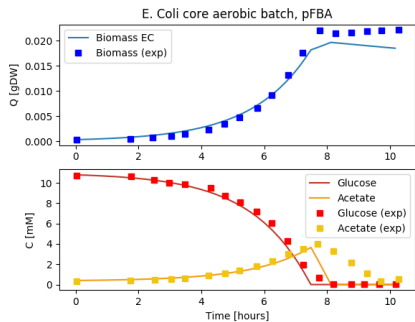

(a)

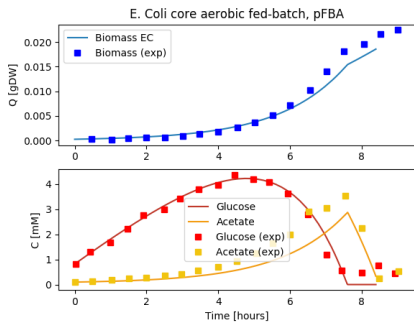

(b)

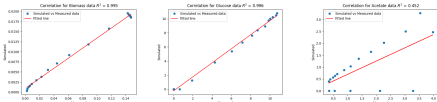

(c) batch, pFBA

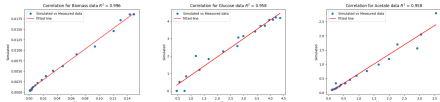

(d) fedbatch, pFBA

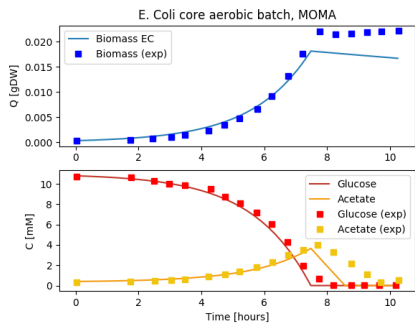

(e)

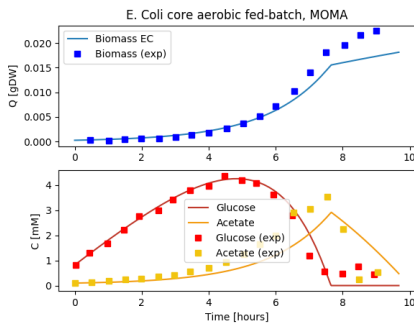

(f)

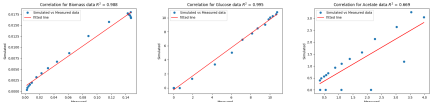

(g) batch, MOMA

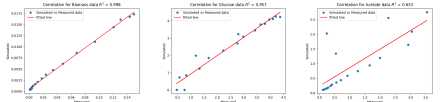

(h) fedbatch, MOMA

Supplement: FIG S1 [file mSystems.00230-18-sf001.pdf]

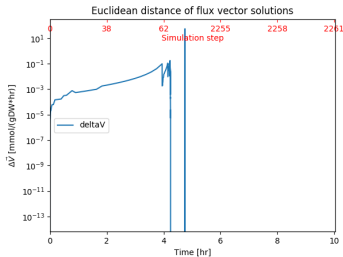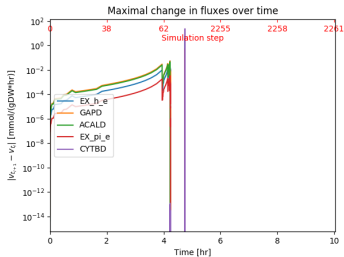

(a) Enjalbert et al., glucose only, pFBA

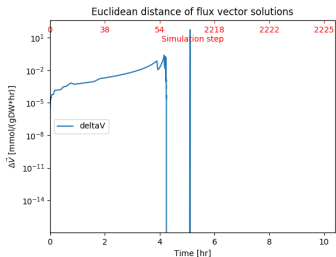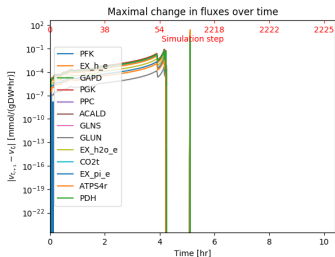

(b) Enjalbert et al., glucose only, MOMA

Supplement: FIG S3 [file mSystems.00230-18-sf003.pdf]

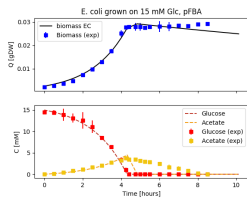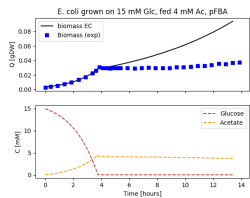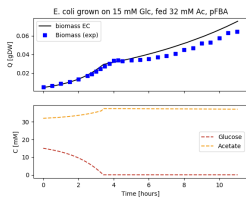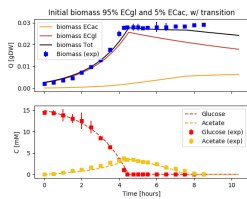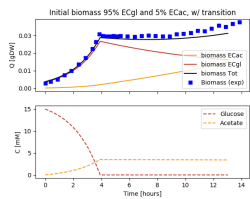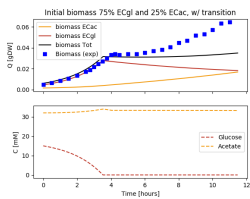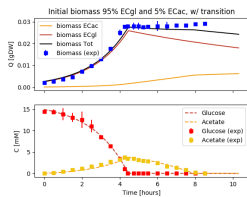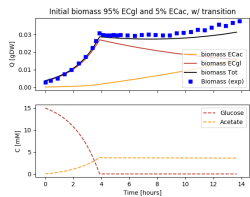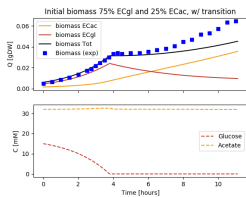

Supplement: FIG S5 [file mSystems.00230-18-sf005.pdf]

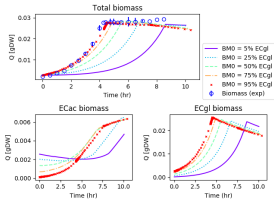

(a)

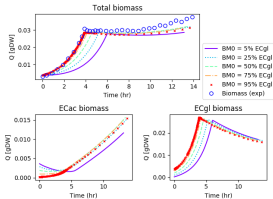

(b)

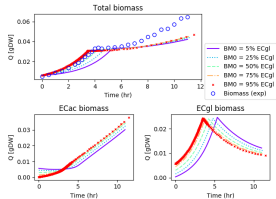

(c)

Supplement: FIG S6 [file mSystems.00230-18-sf006.pdf]

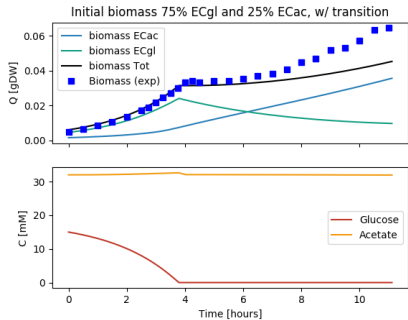

(a)

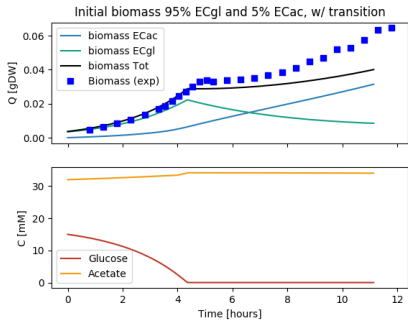

(b)

Supplement: FIG S7 [file mSystems.00230-18-sf007.pdf]
