# Supplementary material for: Emergent Subpopulation Behavior Uncovered with a Community Dynamic Metabolic Model of Escherichia coli Diauxic Growth
Source: mSystems. 2019 Jan 15;4(1):e00230-18. doi: 10.1128/mSystems.00230-18 (PMC6446979; doi:10.1128/mSystems.00230-18)
Supplement: FIG S2 [file mSystems.00230-18-sf002.pdf]

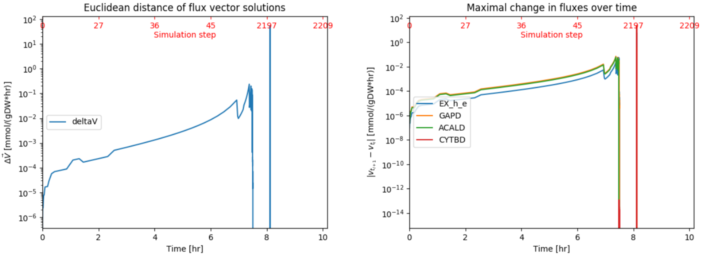

(a) Varma and Palsson, batch, pFBA

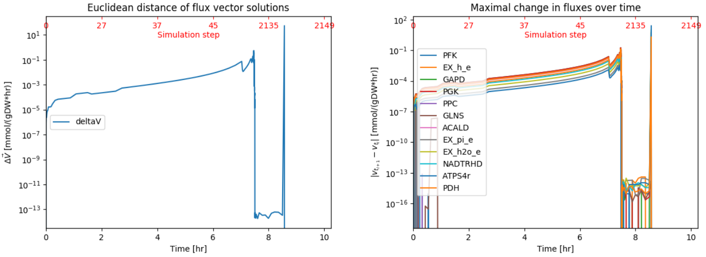

(b) Varma and Palsson, batch, MOMA

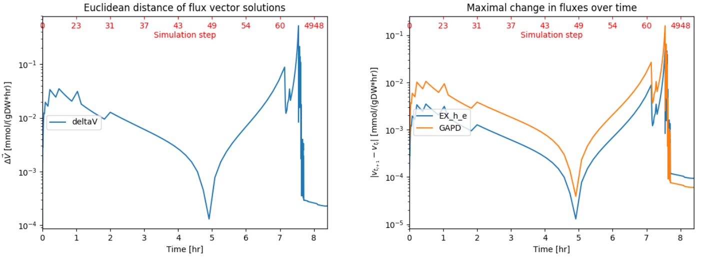

(c) Varma and Palsson, fedbatch, pFBA

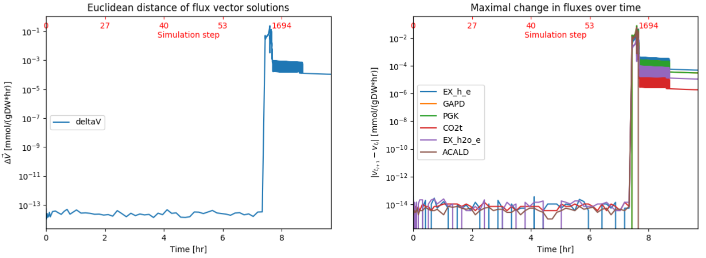

(d) Varma and Palsson, fedbatch, MOMA
