# Supplementary material for: Emergent Subpopulation Behavior Uncovered with a Community Dynamic Metabolic Model of Escherichia coli Diauxic Growth
Source: mSystems. 2019 Jan 15;4(1):e00230-18. doi: 10.1128/mSystems.00230-18 (PMC6446979; doi:10.1128/mSystems.00230-18)
Supplement: TEXT S1 [file mSystems.00230-18-s0001.pdf]

Emergent sub-population behavior uncovered with a community dynamic metabolic model of *Escherichia coli* diauxic growth

Antonella Succurro [1,2]*, Daniel Segrè[3,4] and Oliver Ebenhöh [5,2]

[1] Botanical Institute, University of Cologne, Zülpicher Straße 47b, 50674 Cologne, Germany
[2] CEPLAS (Cluster of Excellence on Plant Sciences), Heinrich-Heine University, Universitätsstraße 1, 40225 Düsseldorf, Germany
[3] Bioinformatics Program and Biological Design Center, Boston University, Boston, MA, USA
[4] Department of Biology, Department of Biomedical Engineering, Department of Physics, Boston University, Boston, MA, USA
[5] Institute of Quantitative and Theoretical Biology, Heinrich-Heine University, Universitätsstraße 1, 40225 Düsseldorf, Germany

* Correspondence: a.succurro@uni-koeln.de (A.S.)

Supplemental Material TEXT S1

Ordinary differential equation model of two subpopulations in constant environment

Let there be two *E. coli* sub-populations, one consuming exclusively glucose G at a rate v_G^- and the other consuming exclusively acetate A at a rate v_A^-. The populations biomass and growth rates are X_G, μ_G and X_A, μ_A respectively, and the growth rates might depend on the substrate availability. X_G produces A at a rate v_A^+. Let us assume that the population X_G can transit to the population X_A with a rate $\psi(A)$, dependent on acetate concentration, and the population X_A can transit to the population X_G with a rate $\phi(G)$, dependent on glucose concentration. Both transitions have an efficiency $\epsilon \in [0, 1]$. The dynamics of such system can be represented by the following ordinary differential equation system:

$$\frac{dX_A}{dt} = \mu_A(A)X_A - \phi(G)X_A + \epsilon\psi(A)X_G \tag{1}$$
$$\frac{dX_G}{dt} = \mu_G(G)X_G - \psi(A)X_G + \epsilon\phi(G)X_A \tag{2}$$
$$\frac{dG}{dt} = -v_G^- X_G \tag{3}$$
$$\frac{dA}{dt} = v_A^+ X_G - v_A^- X_A. \tag{4}$$

Assuming an environment where G and A levels do not change (Eqs. 3 and 4 equal 0), with constant maximal growth rates, we are interested in studying if such system evolves towards a constant population ratio $\Gamma = X_G/X_A$. The ODE system we want to solve is therefore simply:

$$\frac{dX_A}{dt} = \mu_A X_A - \phi X_A + \epsilon\psi X_G \tag{5}$$
$$\frac{dX_G}{dt} = \mu_G X_G - \psi X_G + \epsilon\phi X_A. \tag{6}$$

Under the condition $d\Gamma/dt = 0$, the solution to the ODE system of Eqs. 5–6 is then reduced to the equation:

$$-\epsilon\psi\Gamma^2 + (\mu_G - \mu_A - \psi + \phi)\Gamma + \epsilon\phi = 0 \tag{7}$$

In the case of $\epsilon = 0$ (*i.e.* ψ and ϕ correspond to death rates) the solutions read:

$$\mu_G - \psi = \mu_A - \phi \tag{8}$$
$$\Gamma = 0. \tag{9}$$

Let us assume positive values of $\epsilon \leq 1$. The solution is then:

$$\Gamma = \frac{\mu_G - \mu_A - \psi + \phi}{2\epsilon\psi} \pm \frac{\sqrt{(\mu_G - \mu_A - \psi + \phi)^2 + 4\epsilon^2\psi\phi}}{-2\epsilon\psi} \tag{10}$$

In order to investigate the relation between the parameters ϵ, ψ and ϕ and the Γ values at which the population relative concentration stays constant, we distinguish three interesting scenarios: : (a) Glucose only: $\mu_G = 0.57$ hr^{-1}, $\mu_A = 0$ hr^{-1}; (b) Acetate only: $\mu_G = 0$ hr^{-1}, $\mu_A = 0.23$ hr^{-1}; (c) Mixed: $\mu_G =$

$0.57\ \mathrm{hr}^{-1}$, $\mu_A = 0.23\ \mathrm{hr}^{-1}$. The growth rate values are the ones measured experimentally by Enjalbert *et al.* Scenario (a), where only growth on glucose is possible, implicitly assumes that the acetate eventually secreted by X_G is immediately flushed out and cannot sustain the growth (following also the original assumption $dG/dt = dA/dt = 0$). In environment (b) only acetate is present, while under condition (c) both acetate and glucose can sustain the populations growth. These conditions could easily be reproduced in single cell flow cytometry devices.

We perform (ψ, ϕ) parameters scan for $\epsilon = 0.9$ and for each condition, calculating the solutions of Eq. 10. Only the negative root gives a positive (and hence meaningful) Γ value. In conditions (a) and (c), where the faster growth on glucose is possible, we assume $\log \Gamma > 0$, while in condition (b), where only growth on acetate is possible, $\log \Gamma < 0$. Table S1 reports the results of scanning ψ (ϕ) while fixing ϕ (ψ) to minimal and maximal values. A minimal transition value corresponds to a constant noise term that is present independently on the medium. A maximum transition values correspond to the situation where high concentrations of the corresponding carbon source are present in the medium. Fixing $\psi = 0.2$, $\phi = 0.2$, $\epsilon = 0.9$, $X_A(0) + X_G(0) = 0.001$, $\Gamma_0 = 0.999$, we simulate the system of Eqs. 5–6 varying, once at a time, $\epsilon, \psi, \phi, \Gamma_0$ between 0 and 1.
