# Supplementary material for: Emergent Subpopulation Behavior Uncovered with a Community Dynamic Metabolic Model of Escherichia coli Diauxic Growth
Source: mSystems. 2019 Jan 15;4(1):e00230-18. doi: 10.1128/mSystems.00230-18 (PMC6446979; doi:10.1128/mSystems.00230-18)
Supplement: TABLE S1 [file mSystems.00230-18-st001.pdf]

Minimal $\phi = 0.04$ (low glucose)		
ψ	(b) Acetate only	(c) Mixed
0.020	-1.780	2.979
0.041	-1.882	2.234
0.061	-1.973	1.774
0.082	-2.055	1.430
0.102	-2.130	1.148
0.122	-2.199	0.904
0.143	-2.264	0.686
0.163	-2.324	0.486
0.184	-2.380	0.299
0.204	-2.433	0.123

Maximal $\phi = 0.2$ (high glucose)		
ψ	(a) Glucose only	(c) Mixed
0.020	3.715	3.354
0.041	3.000	2.635
0.061	2.574	2.203
0.082	2.264	1.889
0.102	2.019	1.639
0.122	1.815	1.430
0.143	1.639	1.250
0.163	1.483	1.090
0.184	1.343	0.947
0.204	1.215	0.816
0.224	1.098	0.696
0.245	0.988	0.584
0.265	0.886	0.480
0.286	0.789	0.383
0.306	0.698	0.291
0.327	0.611	0.204
0.347	0.528	0.121

Minimal $\psi = 0.04$ (low acetate)		
ϕ	(a) Glucose only	(c) Mixed
0.020	2.729	2.192
0.041	2.768	2.259
0.061	2.804	2.321
0.082	2.840	2.379
0.102	2.874	2.433
0.122	2.906	2.484
0.143	2.938	2.533
0.163	2.969	2.579
0.184	2.998	2.622
0.204	3.027	2.664
0.224	3.055	2.704
0.245	3.082	2.743
0.265	3.109	2.780
0.286	3.134	2.815
0.306	3.159	2.850
0.327	3.183	2.883
0.347	3.207	2.915

Maximal $\psi = 0.2$ (high acetate)		
ϕ	(b) Acetate only	(c) Mixed
0.041	-2.401	0.164
0.061	-1.967	0.297
0.082	-1.651	0.407
0.102	-1.400	0.503
0.122	-1.190	0.587
0.143	-1.009	0.663
0.163	-0.850	0.731
0.184	-0.707	0.794
0.204	-0.577	0.853
0.224	-0.458	0.907
0.245	-0.348	0.959
0.265	-0.246	1.007
0.286	-0.151	1.052
0.306	-0.062	1.095
