# Supplementary material for: Emergent Subpopulation Behavior Uncovered with a Community Dynamic Metabolic Model of Escherichia coli Diauxic Growth
Source: mSystems. 2019 Jan 15;4(1):e00230-18. doi: 10.1128/mSystems.00230-18 (PMC6446979; doi:10.1128/mSystems.00230-18)
Supplement: FIG S8 [file mSystems.00230-18-sf008.pdf]

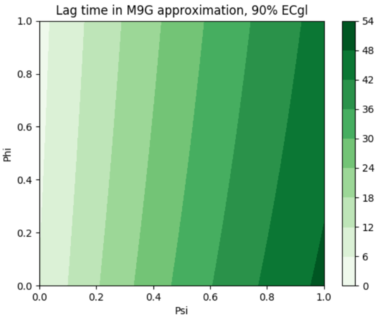

(a) $\psi = \psi_0$

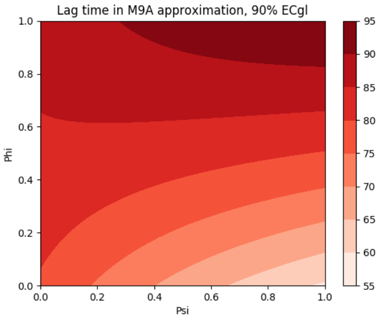

(b) $\phi = \phi_0$

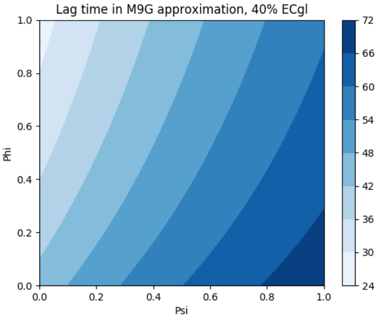

(c) $\psi = \psi_0$

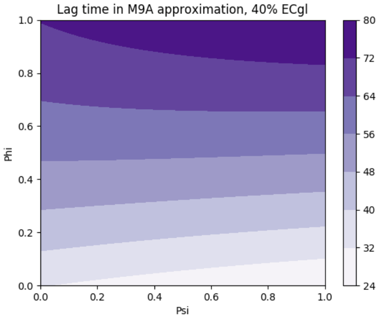

(d) $\phi = \phi_0$

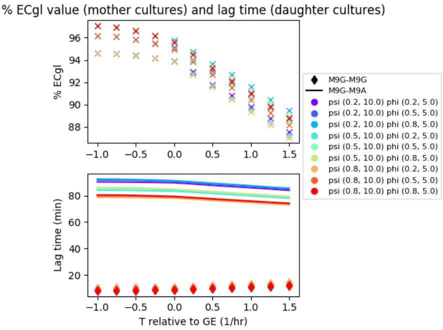

(e) $K^{\psi} = 10$, M9G

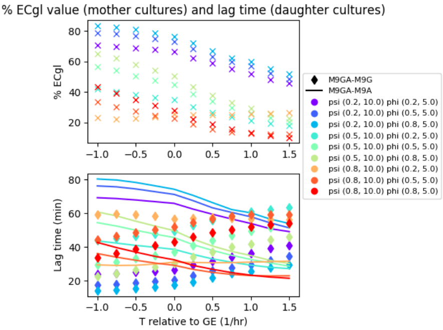

(f) $K^{\psi} = 10$, M9GA

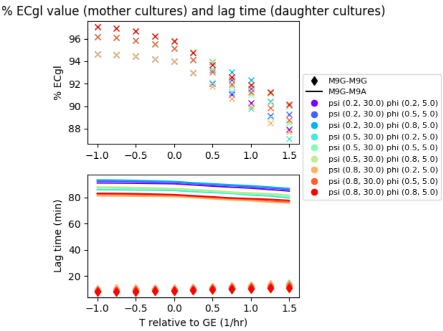

(g) $K^{\psi} = 30$, M9G

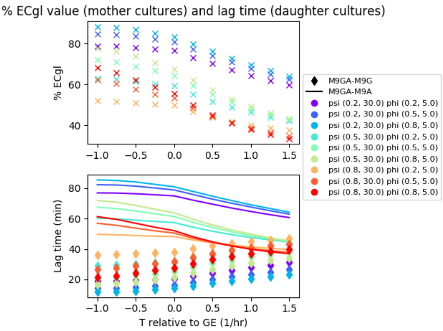

(h) $K^{\psi} = 30$, M9GA
